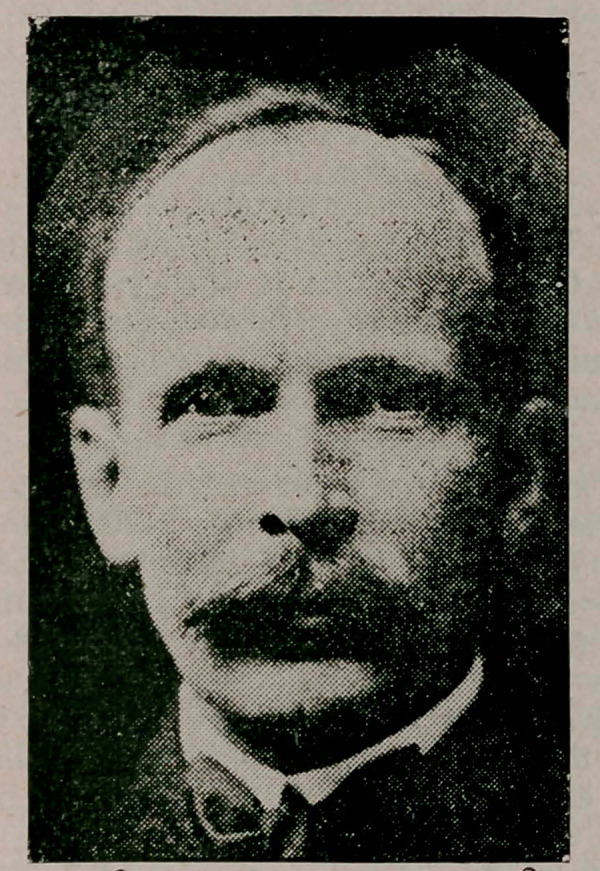# Dr. Eugene A. Smith

**Published:** 1914-09

**Authors:** 


Dr. Eugene A. Smith, Buffalo, 1887, died at his home in Saranac Lake, August 8, aged 49. Dr. Smith was born in Buffalo and graduated from the Central High School in 1883. He graduated with highest honors from the University of Buffalo and served as interne in the Buffalo General Hospital. He engaged in practice with his father, the late James S. Smith whose obituary was published in our issue of March, 1912, later opening a separate office and devoting himself to surgery, being a member of the faculty of Niagara University and subsequently of the University of Buffalo after the merging of the two schools of medicine, and having a service at the Sisters' and other hospitals. Like his father, he developed tuberculosis but did not, as his father did, throw off the disease in middle life, although he succeeded in regaining some measure of health and in pursuing his professional labors for many years. Even as a boy, Dr. Smith was interested in natural sciences. As a man, he united dignity and affability, scholarship and practical ability, a serious nature with geniality, as few men harmonize such contrasting qualities· Like his father, he was interested in military matters. Dr. Smith's military record will be prepared by Lt.-Col. A. H. Briggs, M. D., and will be published in our next issue.

## Figures and Tables

**Figure f1:**